# The neuroprotective potential of curcumin on *T. Spiralis* infected mice

**DOI:** 10.1186/s12906-024-04399-0

**Published:** 2024-02-22

**Authors:** Magda SA Abdeltawab, Iman R. Abdel-Shafi, Basma Emad Aboulhoda, Amal M. Mahfoz, Alshaimaa MR Hamed

**Affiliations:** 1 Department of Medical Parasitology, Faculty of Medicine, Cairo University, Cairo, Egypt; 2 Anatomy and Embryology Department, Faculty of Medicine, Cairo University, Cairo, Egypt; 3 Department of Pharmacology and Toxicology, Faculty of Pharmacy, Modern University for Technology and Information, Cairo, Egypt

**Keywords:** Cerebral trichinosis, Albendazole, Curcumin, Dopamine, Oxidative stress, Cyclooxygenase-2, CD34

## Abstract

**Background:**

*Trichinella spiralis* can affect the brain by inducing inflammatory and vascular changes. Drug management with the antiparasitic drug albendazole can be enhanced by natural compounds such as curcumin. The potential benefit of curcumin as an adjuvant to albendazole in the management of cerebral affection during experimental *T. spiralis* infection was evaluated. Animals received either curcumin 150 mg/Kg, albendazole 50 mg/Kg or a combination of both drugs. Animal groups receiving treatment were compared with infected and non-infected control groups. Blood levels of reduced glutathione (GSH) and dopamine were measured, and brain tissue expression of cyclooxygenase-2 enzyme (COX-2) and CD34 was assessed by immunohistochemistry.

**Results:**

*T. spiralis* infection resulted in a state of oxidative stress, which was improved by albendazole and curcumin. Also, both drugs restored the peripheral dopamine level, which was decreased in infected non-treated mice. Curcumin was also found to be efficient in improving brain pathology and reducing local COX-2 and CD 34 expression.

**Conclusions:**

Inflammatory and pathological changes during neurotrichinosis can be improved by the addition of curcumin to conventional anti-parasitic drugs.

## Background

*Trichinella spiralis* (*T. spiralis*) is a parasitic nematode that affects both animals and humans. Infection by this parasite is known to induce intestinal pathology by the adult stages of the worm, and muscular pathology by its larval stages. Systemic manifestations can occur due to the generalized inflammatory reaction and the excessive immune stimulation associated with larval migration [1, 2]. Severe trichinosis can result in multi-organ involvement including the heart, lungs and brain. Brain affection during trichinellosis can present either as a diffuse cerebral affection or focal neurological deficit, and mortality rates reaching up to 50% have been reported [3]. The pathology of brain affection in neurotrichinosis includes vascular changes such as blood vessel obstruction, vasculitis, thrombosis and hemorrhage, in addition to granulomatous and allergic inflammatory reactions [4].

The immune response to *T. spiralis* infection results in the formation of free radicles, which are released by activated phagocytes. Reactive oxygen species (ROS) target the parasite in both its intracellular and extracellular niches. The resulting oxidative stress, however, is an important contributor to *T. spiralis* induced pathology [5].

Dopamine metabolism contributes significantly to oxidative stress, due to the production of cytotoxic dopamine and dopamine quinones, which harbor highly reactive hydroxyl residues [6]. *T. spiralis* is reported to induce apoptosis of dopaminergic neurons and affect central dopamine levels [2]. Since dopamine does not cross the blood brain barrier, plasma dopamine is of peripheral origin. It is synthesized mainly in sympathetic nerves and is transported to various effector sites inside platelets. It also acts as an autocrine or paracrine messenger and exerts neuroregulatory and immunoregulatory actions [7]. Moreover, peripheral dopamine released from synaptic neurons interferes with mesenchymal cell migration through acting on the dopamine D2 receptor, thus opposing angiogenesis and wound repair [8].

Benzimidazole derivatives are the main anthelminthics used in the management of *T. spiralis* infection. The most famous member of this drug family is albendazole, which disrupts the structure of the parasite by binding to cuticular tubulin [9]. Natural herbs such as curcumin, are effective therapeutic agents against parasitic diseases, including trichinosis [10]. Curcumin, a member of the family *Zingiberaceae*, genus *Curcuma*, contains active polyphenolic curcuminoids, which include diferuloylmethane and curcumin [11]. Curcumin has a rapid intestinal and hepatic metabolism which results in the fecal excretion of 60–70% of oral doses [12]. In addition, intestinal cells conjugate curcumin into the metabolite tetrahydrocurcumin. Despite of its low oral bioavailability, the lipophyllic nature of curcumin favors its transport through the blood brain barrier, making it a promising therapeutic agent in nervous system diseases. It was observed, however, that curcumin efficiently crosses the blood brain barrier in its parent form rather than its conjugated form, which favors the parenteral rather than the oral administration of curcumin [13]. The effects of curcumin include the enhancement of anti-oxidant protein expression, the scavenging of free radicles, and the selective inhibition of cyclooxygenase-2 (COX-2) mRNA, a key mediator of inflammatory pathways [14, 15]. Curcumin also inhibits angiogenesis either directly or by targeting factors such as fibroblast growth factor (FGF), vascular endothelial growth factor (VEGF), and matrix metalloproteinases (MMPs) [16]. Angiogenesis is a crucial hallmark of *T. spiralis* infection, which induces the up-regulation of VEGF [17]. Microvessel density (MVD) reflects the degree of angiogenesis and is determined by the intercapillary space, which is influenced by the balance between anti- and pro-angiogenic factors [18]. MVD can be assessed by CD34 and CD105, both of which are endothelial markers [19]. CD34 is a highly glycosylated sialomucin located on the surface of cells. It is an important effector in cell adhesion and trafficking [20].

In the current study, we studied the effect of albendazole and the benefit of curcumin as an adjuvant therapy on experimental neurotrichinosis regarding angiogenesis, inflammation, oxidative stress and peripheral dopamine level. GSH was measured as an indicator for oxidative stress, while dopamine was measured due to its impact on immnue cell function, trafficking and angiogenesis. Brain tissue expression levels of COX-2 and CD34 were assessed to evaluate the effect of these therapeutic agents on inflammation and angiogenesis.

## Methods

### Parasite and animals

The *T. spiralis* strain was provided from the department of Medical Parasitology, Faculty of Medicine, Cairo University. The experiment was conducted in the animal house of the Faculty of Pharmacy, Modern University for Technology and Information, Cairo, Egypt.

Adult male Swiss albino mice, each weighing 25–30 g, were bred under standard conditions (12-hour light/dark cycle, 25 ± 2℃ room temperature, and ad libitum access to water and standard pellet diet). Each mouse received an infective dose of 200+/- 10 *T. spiralis* larvae by gastric feeding, after being starved for 12 h [21].

### Drugs

Both drugs were administered intra-peritoneally (IP) after dissolution in Tween 80 and dilution in normal saline [22]. *Curcuma longa* -derived pure curcumin powder was purchased from Sigma-Aldrich, USA, assay: ≥ 65% curcumin (HPLC), [CAS: 458-37-7; MW: 368.38 g/mol; C21H20O6]. Each mouse received a daily dose of 150 mg/kg [23]. Albendazole powder (Sigma pharmaceutical industries – Egypt) was given at a daily dose of 50 mg/kg [24–28].

### Study groups

Five animal groups of 7 mice each were included in this study. All animals were sacrificed after 4 weeks of the start of the experiment marked by the infection of animals with *T. spiralis* larvae.

#### Group 1

Comprised the control non-infected mice.

#### Group 2

Comprised the infected non-treated mice. Animals received saline and Tween 80 1 week PI for 3 weeks.

#### Group 3

Comprised infected mice receiving curcumin starting 1 week PI for 3 weeks.

#### Group 4

Comprised infected mice receiving albendazole starting 1 week PI for 3 weeks.

#### Group 5

Comprised infected mice receiving albendazole combined with curcumin starting 1 week PI for 3 weeks.

### Sample collection and experimental procedures

Mice were euthanized by cervical dislocation under anaesthesia. After completion of the drug regimen, muscle larval count was determined per gram muscle tissue by digestion of the recti abdominis muscles in 1% HCl and 1% pepsin followed by repeated sieving and microscopic counting of larvae after sedimentation [21]. Blood samples were collected to estimate reduced glutathione (GSH) and dopamine levels. Brain tissue was preserved for histopathological and immunohistochemical studies.

### Colorimetric assessment of plasma glutathione (GSH) levels

Plasma GSH level was evaluated as described in the reduced Glutathione kit (BIOACTIVIA DIAGNOSTIC test kit, Egypt), which is based on the reduction of 5,5’ dithiobis (2-nitrobenzoic acid) (DTNB) with glutathione (GSH) to produce a yellow compound. The reduced chromogen showed a direct proportional relation to the GSH concentration. Its absorbance was measured at 405 nm [29]. GSH level was expressed in mg/dl, where the reference value provided in the kit was 4 mg/dl.

### Measurement of dopamine level in plasma by enzyme linked immunosorbent assay (ELISA)

Plasma dopamine level was estimated according to the dopamine ELISA kit (Labor Diagnostika Nord GmbH &Co.KG test kit, Germany). Dopamine was detected by an anti-rabbit IgG-peroxidase conjugate. This was achieved by using 3,3’,5,5’-tetramethylbenzidine (TMB) as a substrate. The reaction was observed at 450 nm. Absorbance was compared with a reference curve prepared with known standard concentrations. Dopamine level was expressed in pg/ml [30].

### Histopathological examination of the brain

Fixation of brain tissue was done in 10% formalin. Tissue samples were then dehydrated, cleared, and embedded in paraffin blocks. These were cut into 5 μm thick sections, mounted and stained with haematoxylin and eosin (Hx&E). Examination for histopathological changes was done in 10 high power fields (HPF, x 400) in each tissue Sects [31, 32].

### Assessment of COX-2 and CD34 expression by immunohistochemical (IHC) staining

Primary anti-cyclooxygenase-2 antibody (COX-2) (ab15191, 1/100, rabbit polyclonal antibody, Abcam, USA) and anti-CD34 antibody (ab185732, 1/50, rabbit polyclonal antibody, Abcam, USA, species specificity including mice) were used for immunohistochemical staining. Heat-mediated antigen retrieval and standard labeled streptavidin–biotin immunoenzymatic antigen detection procedures were performed according to Aboulhoda & Abd el Fattah [33]. Counterstaining of tissue sections with Mayer’s hematoxylin was performed. Immunostaining of mouse liver tissue provided the positive control according to the manufacturer’s protocol, while the negative control was obtained by the omission of incubation with the primary antibody in the automated staining protocol.

The count of CD34 immunoreactive cells and the area percentage of COX-2 immunohistochemical expression were assessed via the optical ImageJ image analyzer Computer software program (ImageJ analysis system, version 1.53 MD, USA) affixed to a computer in six fields (x40) with a standard measuring frame of 85,550 µm^2^ using a Leica Qwin 500 Ltd microscope digital camera (Leica Microsystems, Wetzlar, Germany).

### Statistical analysis

Data was expressed as means and standard deviations (SD). Group comparisons were done using analysis of variance (ANOVA), followed by Tukey Kramer post-hoc test. P-values < 0.05 were considered statistically significant.

## Results

### Effect of drug regimen on larval muscle count

Muscle larval count in the infected non-treated group was 17.6 ± 0.9/gm. Curcumin administration lead to a significant reduction in larval load (13.5 ± 1.1gm; *p* < 0.05). The most efficient reduction in the number of larvae was observed in mice receiving albendazole only (7.7 ± 0.8/gm) and combined albendazole and curcumin (7.3 ± 0.8/gm) (*p* < 0.05 when compared to non-treated and curcumin-treated mice).

### Effect of the different drug regimens on plasma reduced glutathione level (GSH) expressed in mg/dl

GSH level in the negative control group was 11.29 ± 0.88 mg/dl. Plasma level of GSH was 4.67 ± 0.49 md/dl in the infected non-treated group, which was significantly lower than the rest of the study groups. Consumption of GSH occurs during oxidative stress. Plasma samples from albendazole-treated mice showed a GSH level of 13.03 ± 2.14 mg/dl. GSH levels in mice receiving a combination of albendazole and curcumin had a mean value of 12.58 ± 1.97 mg/dl. Both groups showed significantly higher GSH levels as compared to levels from mice receiving curcumin alone (9.15 ± 0.29 mg/dl).

### Effect of different drug treatments on plasma dopamine expressed in pg/ml

The mean value of dopamine levels in the negative control group was 42.45 ± 0.36 pg/ml. Dopamine levels in the plasma of infected non-treated mice had a mean level of 32.25 ± 0.89 pg/ml, which was significantly lower than the dopamine level observed in the rest of the study groups (*P* < 0.05). Dopamine level in mice receiving curcumin was 40.65 ± 0.25 pg/ml, which was significantly lower than that detected in samples from albendazole-treated mice (42.81 ± 1.03 pg/ml), and mice receiving dual curcumin and albendazole therapy (42.79 ± 0.57 pg/ml) (*P* < 0.05).

### Histopathological examination of the brain tissue

**Fig. 1:**
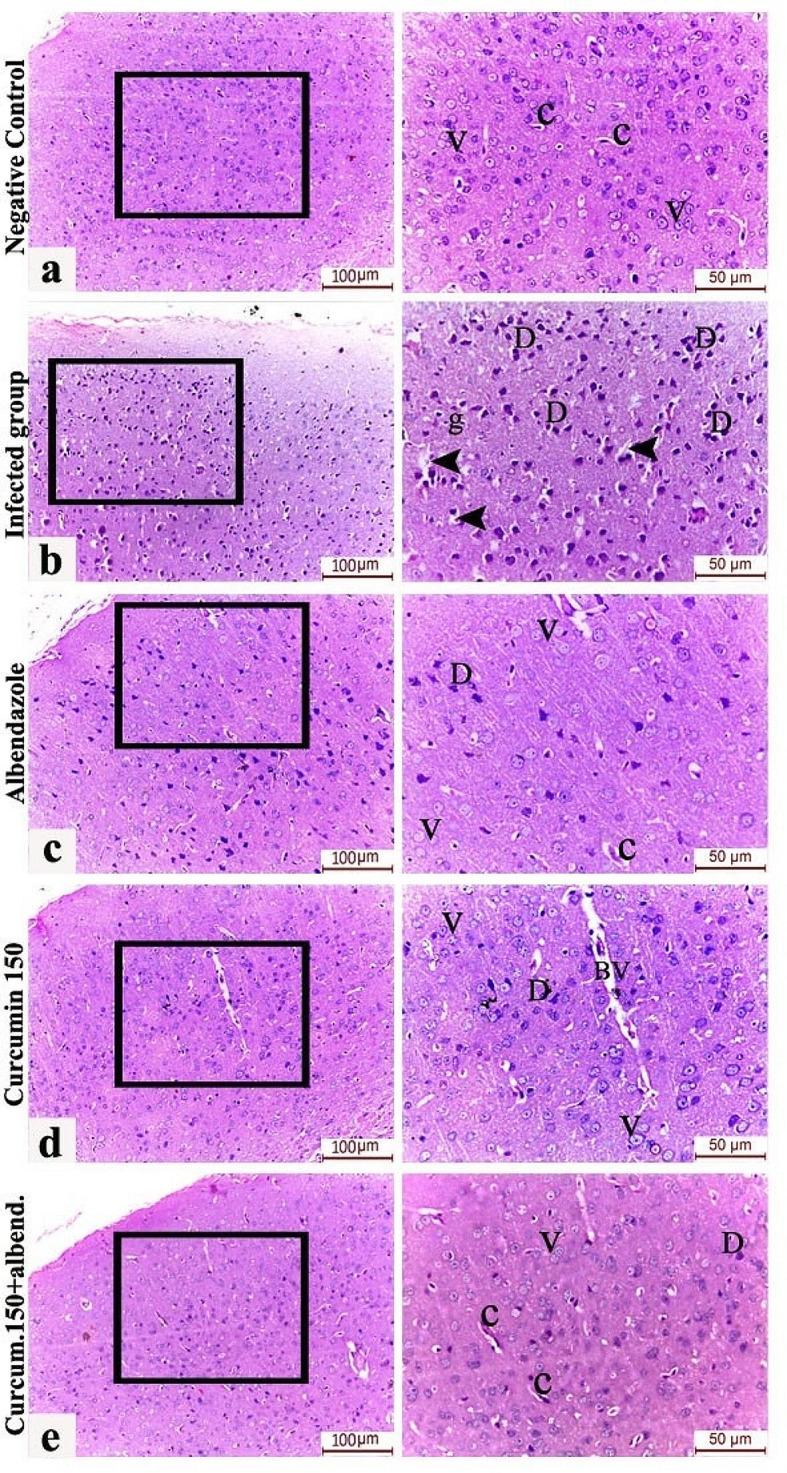
H&E-stained sections of the brain cortical tissue of the various study groups. [**a**] Group 1 (non-treated non-infected group) showing normal brain tissue. [**b**] Group 2 (infected non-treated group) showing numerous darkly-degenerated neurons with pyknotic, shrunken deeply-stained nuclei (D), perineuronal halos (arrow heads) and multiple neuroglia (g); [**c**] Group 3 (albendazole-treated group); [**d**] Group 4 (curcumin-treated group); [**e**] Group 5 (dual curcumin and albendazole therapy). All treated groups showed improvement in the cerebral cortical architecture (being most prominent in the dual therapy groups), where most of the neurons are seen exhibiting rounded to oval vesicular nuclei with prominent nucleoli (V). Very few dark neurons (D) can be observed. (Abbreviations: D: Darkly-degenerated neurons, V: vesicular neurons, g: neuroglial cells, c: capillaries, BV: Blood vessel with widened perivascular space (Virchow-Robin space).

Numerous darkly degenerated neurons with pyknotic, shrunken and deeply stained nuclei and peri-neuronal halos were observed in the cerebral cortex of the *Trichinella spiralis*-infected group. Improvement of these pathological changes was observed in all study groups, being most prominent in the combined albendazole and curcumin treatment group (Fig. 1).

### Effect of the different drug regimens on COX-2 expression in brain tissue

**Fig. 2:**
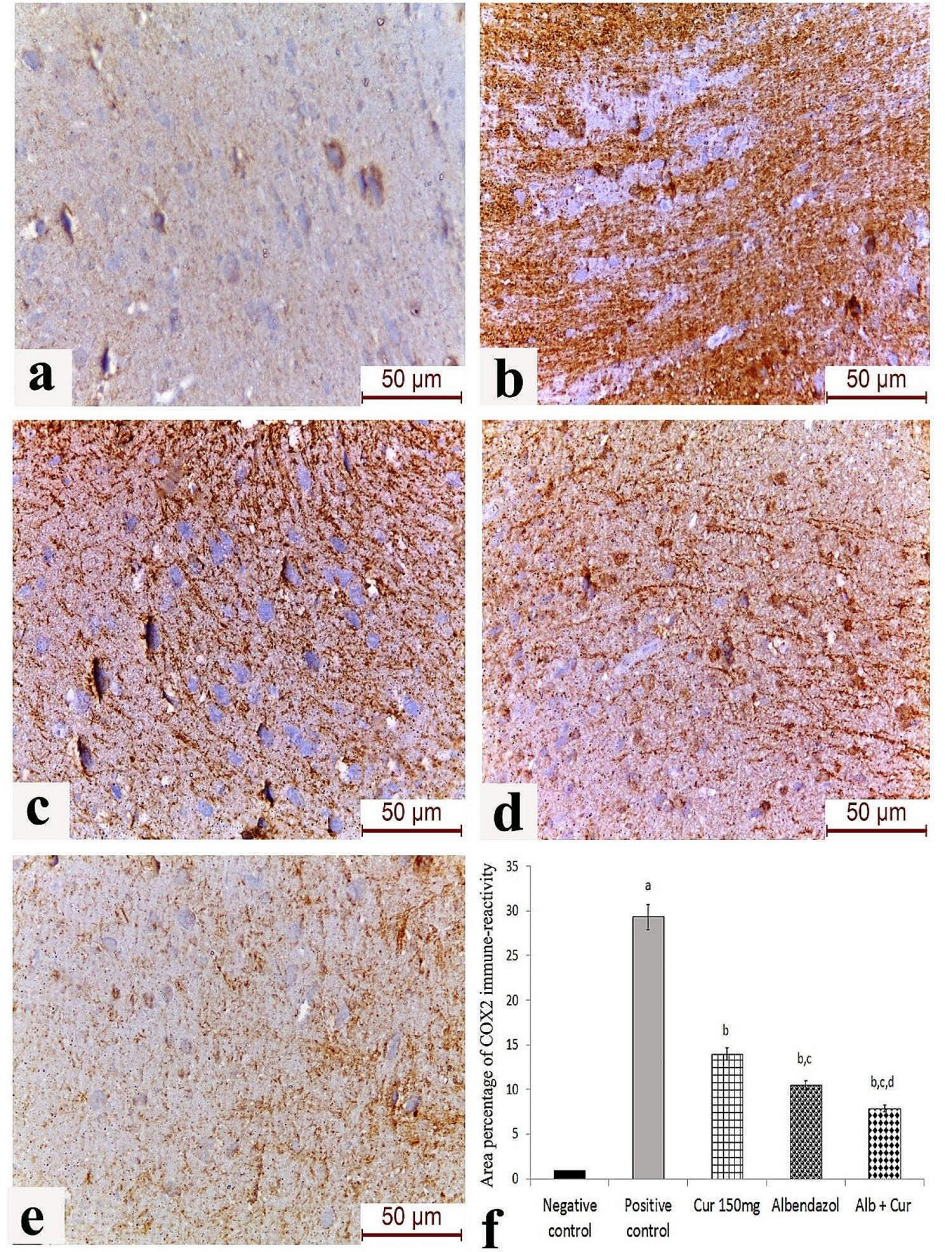
Expression of COX-2 in the cerebral cortex by IHC: [**a**] Group 1 (non-infected group); [**b**] Group 2 (infected non-treated group); [**c**] Group 3 (albendazole therapy group); [**d**] Group 4 (curcumin therapy group); [**e**] Group 5 (dual curcumin and albendazole therapy group). Strong positive COX-2 immune-reactivity in the brain tissue of infected non-treated mice and moderate immune-reactivity in the albendazole and curcumin-treated groups can be observed. Weak COX-2 expression is noticed in the combined dual curcumin and albendazole therapy group; scale bar 50 μm. (**f**) Area percentage of COX-2 immunohistochemical expression in the different study groups; a: Significant versus negative control, b: Significant versus positive control, c: Significant versus curcumin group, d: Significant versus Albendazole group at *P* < 0.05 using Tukey Kramer post-Hoc comparison test

Marked expression of COX-2 was detected in the cerebral cortex of *T. spiralis*-infected mice. Moderate cytoplasmic expression of COX-2 was observed in the albendazole-treated group, while weak cytoplasmic expression with statistically-significant reduction in the area percentage of COX-2 immunoreactivity (*p* < 0.05) was noticed in the dual curcumin and albendazole therapy group (Fig. 2).

### Effect of the different drug regimens on CD34 expression in brain endothelium

**Fig. 3:**
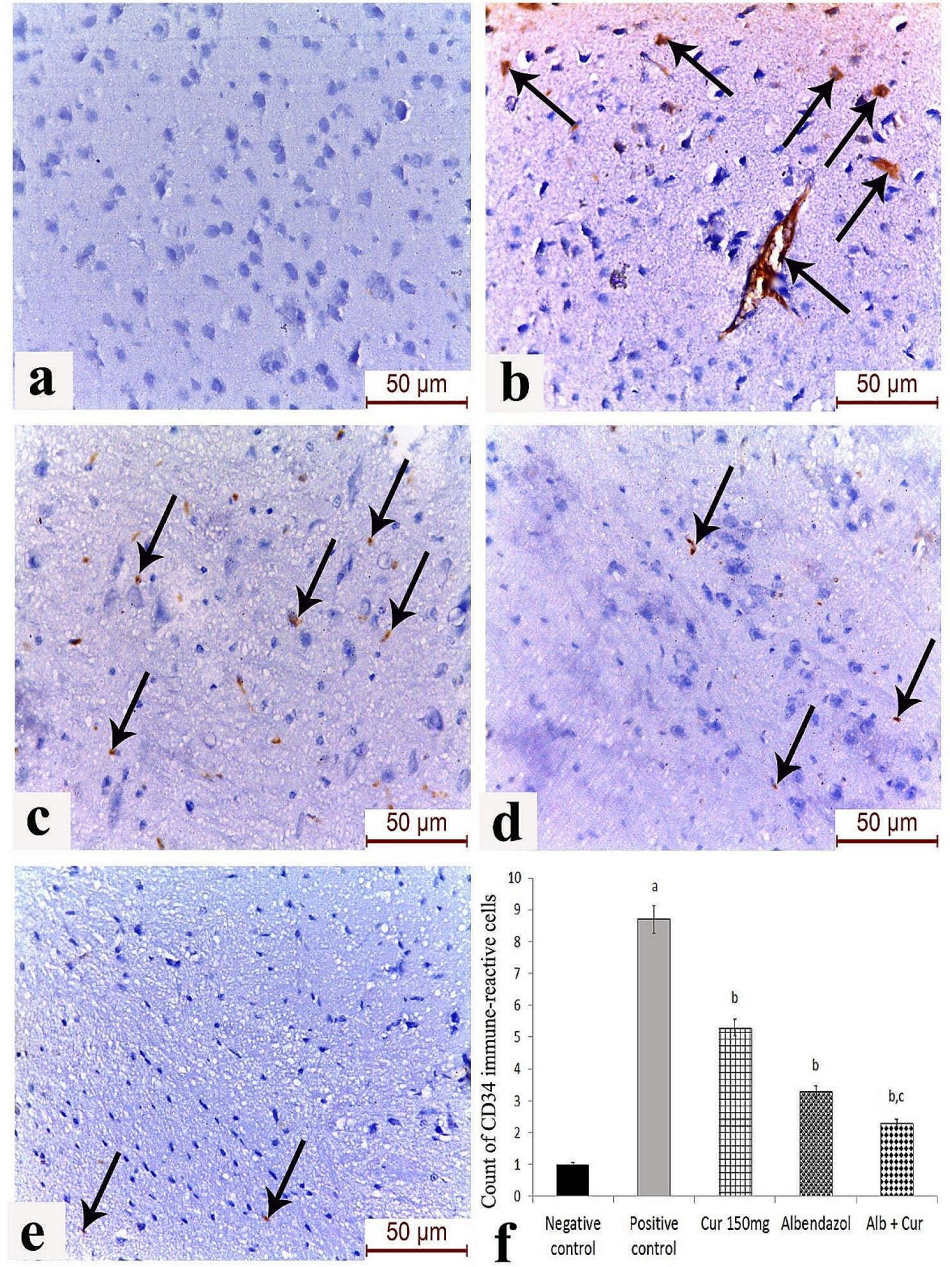
CD34 immune-expression in the cerebral cortex by IHC: [**a**] Group 1 (non-infected group); [**b**] Group 2 (infected non-treated group); [**c**] Group 3 (albendazole-treated group); [**d**] Group 4 (curcumin-treated group); [**e**] Group 5 (dual curcumin and albendazole therapy group). Strong positive CD34 protein expression can be observed in the brain endothelium and inflammatory cells of the infected group. Mild to moderate expression is observed in the curcumin-treated group, while moderate protein expression can be noticed in the albendazole-treated group. Very few inflammatory cells weakly expressing CD34 expression can be observed in the dual curcumin and albendazole therapy group; scale bar 50 μm. [**f**] Count of CD34 immune-reactive cells in the different study groups; a: Significant versus negative control, b: Significant versus positive control, c: Significant versus curcumin 150 group at *P* < 0.05 using Tukey Kramer post-Hoc comparison test. at *P* < 0.05 using Tukey Kramer post-Hoc comparison test

A strong expression of CD34 was observed in the brain endothelium and inflammatory cells of the *T. spiralis*-infected group. Mild to moderate expression was observed in the curcumin-treated group, while moderate protein expression was noticed in the albendazole-treated group. Statistically-significant reduction in the count of CD34 immunoreactive cells (*p* < 0.05) was observed in the combined curcumin and albendazole therapy group (Fig. 3).

## Discussion

Herbal remedies, including the *Curcuma longa* extract curcumin, show potent anti-parasitic effects [11, 21, 34]. Curcumin was found to be effective in reducing the parasite burden during *T. spiralis* infection as demonstrated by Elguindy et al. [35]. The reduction of intestinal worm count and muscle larval count was associated with increased expression of nuclear factor kappa B (NF-κB), an important immune regulator during infection. Curcumin was also found to have neuroprotective effects mediated through its antioxidant and anti-inflammatory capacities. In addition, it modulates central neurotransmitters and prevents neural apoptosis and has thus been studied as a candidate agent against a variety of neurodegenerative disorders [36]. Moreover, it has anti-protein aggregation properties, which protect the brain against amyloid degenerartion [37]. Curcumin also as acts as an epigenetic regulator of cell signaling pathways that are involved in cellular proliferation and death. It enhances the effects of Ach by potentiating the function of its receptor through its effect on Calcium fluxes. Moreover, it protects iron-induced dopaminergic neuronal damage observed during Parkinson’s disease by its iron-chelating properties [38].

*Trichinella* infection of the CNS is a serious complication that results in focal neurological affection or diffuse pathology [39]. Autopsy and biopsy studies showed various pathological findings including infiltrates of endothelial cells, lymphocytes, eosinophils and glial cells, in addition to cerebral hemorrhage and meningeal congestion [3]. In the current research, we studied the effect of curcumin and albendazole on the inflammatory response and local brain tissue expression of COX-2 and CD34 expression in *T. spiralis*-infected mice. We also studied systemic factors affecting the immune response such as peripheral oxidative stress and dopamine level.

Histopathological sections of the brain of infected non-treated mice showed cortical changes including numerous darkly degenerated neurons with pyknotic, shrunken deeply stained nuclei and peri-neuronal halos. Improvement of the cerebral cortical architecture occurred in all drug-treated groups and was most notable in the combined albendazole and curcumin regimen, where most of the neurons exhibited rounded to oval vesicular nuclei with prominent nucleoli. In addition to the histopathological changes, blood GSH level was significantly decreased in the infected non-treated group. GSH is the most important low molecular weight antioxidant produced intracellularly [40]. Curcumin administration was effective in improving GSH levels, however, better results were obtained when it was combined with albendazole, and the highest effect was achieved when albendazole was given alone. In a study by Saad et al. [2], *Trichinella* infection was associated with increased plasma levels of pro-inflammatory cytokines and advanced oxidative protein products. Local brain pathology was characterized by tissue degeneration, neural apoptosis, especially of cortical dopaminergic neurons, with decreased tyrosine hydroxylase activity and brain dopamine levels. These findings were similar to pathological changes occurring in an experimental Parkinson’s disease model developed by the authors.

Pathological changes during parasitic infections are in part the result of immune-mediated oxidative damage. Free radicles generated by immune cells contribute to their cytotoxic effect against parasites [41]. Increased antioxidant enzyme activity was reported during experimental murine trichinosis. Encysted *T. spiralis* larvae elicit the release of reactive oxygen species (ROS) by phagocytes, which explains the exaggerated action of antioxidant enzymes during the muscular phase of infection [5]. The present study results demonstrated a significant reduction in the plasma level of GSH in infected nontreated mice. The increase of GSH level after curcumin administration can be explained by both the antioxidant effect of the drug and the decrease in worm burden. Curcumin was reported to improve hepatic GSH content and reduce hepatic oxidative stress [42]. As for albendazole, it has been shown to induce the generation of reactive oxygen and nitrogen species, which is suggested to be one of the mechanisms underlying its anthelminthic activity [43]. The increase in GSH level after albendazole administration can therefore be attributed to the decrease in worm burden.

The antioxidant capacity of curcumin was reported to be beneficial in the management of both protozoal and helminthic infections [15, 41–45]. Likewise, the antioxidants resveratrol and selenium proved beneficial in the management of experimental trichinosis [46, 47]. The cerebral tissue is particularly sensitive to oxidative stress due to its high oxygen consumption and richness in fatty acids that form lipid peroxides and iron that can generate hydroxyl residues upon oxidation. In addition, the antioxidant capacity of the brain is low as compared to other organs. Hence, peripheral biomarkers of oxidative stress have been associated with aging and neurodegenerative disorders such as Alzheimer’s disease [48].

Since the presence of *Trichinella* larvae in the CNS is not a consistent finding in cases of neurotrichinosis [3], we included systemic factors such as oxidative stress and dopamine level in our study to explore the link between infection-induced systemic biochemical changes and brain pathology. As dopamine does not cross the blood brain barrier, plasma dopamine is only of peripheral source, originating to a great extent from sympathetic neurons. The low plasma levels of dopamine previously intrigued researchers into the assumption that plasma dopamine serves only as a precursor for norepinephrine [49]. However, emerging studies have reported several important functions of peripheral dopamine such as regulation of recruitment and migration of immune cells and mesenchymal cells, regulation of glucose homeostasis and body weight, and control of angiogenesis. In addition, dopamine metabolism is an important contributor to oxidative stress [50, 51].

We also assessed COX-2 expression in brain tissue during *T. spiralis* infection and the effect of conventional albendazole therapy and adjuvant curcumin administration on its expression level by IHC. COX-2 expression was highest in brain sections from infected non-treated mice. Both albendazole and curcumin induced a significant decrease in local COX-2. The lowest COX-2 expression level was observed in mice receiving both albendazole and curcumin. El-Aswad et al. [52] investigated the expression of COX-2 in muscle tissue of *T. spiralis*-infected mice in addition to measuring serum levels of IL-23, IFN-γ, IL-4 and IL-10 cytokines at different time points PI. The authors observed an enhanced expression of serum IL-23 and local COX-2 in the infected muscles peaking on the 35th day PI. Additionally, a mixed Th1/Th2 immune response was detected with a predominance of Th2 cytokines towards later stages of infection. Under physiological conditions, COX-2 enzyme plays an important role in several brain functions such as functional hyperemia, synaptic transmission, anxiety and behaviour, and memory consolidation [53, 54]. Overexpression of COX-2 enzyme has been implicated in the pathogenesis of neurodegenerative disorders through the production of prostaglandin E2 (PGE2), which leads to the release of mediators of apoptosis by microglia. Moreover, neuronal cell death can occur under the effect of cytotoxic reactive oxygen species, such as dopamine quinone, which are produced by the catalytic action of COX-2 [55]. Besides being involved in inflammation, COX-2 was positively correlated with the expression of CD34 in hepatocellular carcinoma, denoting its potential implication in tumour angiogenesis [56]. We have studied the role of angiogenesis in *T. spiralis*-induced brain pathology by evaluating the local expression of CD34 on the surface of endothelial cells in histopathological sections of the brain. Infected non-treated mice showed a significantly elevated level of CD34 expression. Significant reduction of CD34 expression was recorded in mice receiving curcumin either alone or in combination with albendazole. *T. spiralis* up-regulates VEGF by stimulating thymosin β4 [17, 57]. In Ibrahim et al. [58] study on experimental muscular trichinosis, enhanced CD34 expression in myoendothelial cells was observed around the encysted larvae, which was subsequently reduced following administration of artemether. While in Fu et al. [59] research, curcumin was reported to interfere with VEGF – VEGF receptor 2 signaling pathway resulting in reduced VEGF expression. Angiogenesis is not just crucial for nurse cell formation in muscular trichinosis, it is a prominent feature in intestinal inflammation proposing an equally important role in intestinal trichinosis. Angiogenesis is essential for oxygen and nutrient supply, cell recruitment and tissue healing. For instance, an increase in MVD has been described in inflammatory bowel diseases [60].

Curcumin has been demonstrated as an effective treatment, and/or adjunct option against a number of parasitic infections [15, 61, 62]. Curcumin seems to provide an anti-inflammatory and vasculo-protective effect. The anti-inflammatory action is most likely due to its regulatory effect on the transcription and post-transcription of cyclooxygenase and lipoxygenase enzymes that mediate inflammatory processes [63–65].

We have faced several challenges during the design of this experiment. For example, experimental models of neurotrichinosis are not abundant in literature, let alone researches investigating specifically the effects of curcumin and albendazole during cerebral murine *Trichinella spiralis* infection. In addition, curcumin has a rapid intestinal and hepatic metabolism which results in the fecal excretion of 60–70% of oral doses [12]. Moreover, intestinal cells conjugate curcumin into the metabolite tetrahydrocurcumin. Despite of its low oral bioavailability, the lipophyllic nature of curcumin favors its transport through the blood brain barrier, making it a promising therapeutic agent in nervous system diseases. It was observed, however, that curcumin efficiently crosses the blood brain barrier in its parent form rather than its conjugated form [13]. Additionally, nervous system involvement during trichinosis occurs as early as 2 weeks post-infection during the stage of larval migration even before their establishment in skeletal muscles [66]. Since there was no previous clear model in literature to follow, we tried to tailor our own experimental model to achieve an effective regimen drug during the proper of time of infection. Our approach was based on similar research involving experimental murine trichinosis, other parasitic brain infections, and studies on the used therapeutic agents.

## Conclusion

Involvement of the central nervous system during *T. spiralis* infection is a serious complication that is often under-diagnosed. It involves the interaction between systemic immune factors and local inflammatory and vascular changes as reflected by the increased expression of COX-2 and CD34. The effect of *T. spiralis* on effectors such as peripheral dopamine is an interesting aspect, due to its impact on immune regulation and oxidative status. Both curcumin and albendazole were found to be effective in improving cerebral pathology, reducing oxidative stress, suppresssing local COX-2 expression and inflammation, and angiogenesis. Finally, it can be a valuable supplement combined to conventional anti-parasitic drugs to reduce brain inflammation, and improve disease outcome.

## Data Availability

The datasets used and/or analyzed during the current study are available from the corresponding author upon reasonable request.
